# Supplementary material for: Tackling public health data gaps through Bayesian high-resolution population estimation: A case study of Kasaï-Oriental, Democratic Republic of the Congo
Source: PLOS Glob Public Health. 2025 Sep 4;5(9):e0005072. doi: 10.1371/journal.pgph.0005072 (PMC12410768; doi:10.1371/journal.pgph.0005072)
Supplement: S1 Table — The table provides an overview of the gridded geospatial datasets tested in the population count model. The Name column provides the dataset name, Description a succinct description, Year indicates the reference year, Summary outlines the type of processing applied, and Source identifies the dataset origin. All datasets have been processed by WorldPop at the University of Southampton. (PDF) [file pgph.0005072.s001.pdf]

S1 Table. Gridded Geospatial Datasets. The table provides an overview of the gridded geospatial datasets tested in the population count model. The **Name** column provides the dataset name, **Description** a succinct description, **Year** indicates the reference year, **Summary** outlines the type of processing applied, and **Source** identifies the dataset origin. All datasets have been processed by WorldPop at the University of Southampton.

Name	Year	Description	Summary	Source
cod_bld_count_202406 17_unpublished	2024	Building Count	Sum	Center for Integrated Earth System Information (CIESIN)
cod_bld_count_202406 17_unpublished_cov3x 3_tif_masked_tif_lzw	2024	Building Count	Focal Sum (3x3)	Center for Integrated Earth System Information (CIESIN)
cod_bld_count_202406 17_unpublished_cov5x 5_tif_masked_tif_lzw	2024	Building Count	Focal Sum (5x5)	Center for Integrated Earth System Information (CIESIN)
cod_bld_count_202406 17_unpublished_cov7x 7_tif_masked_tif_lzw	2024	Building Count	Focal Sum (7x7)	Center for Integrated Earth System Information (CIESIN)
cod_bld_count_202406 17_unpublished_cov9x 9_tif_masked_tif_lzw	2024	Building Count	Focal Sum (9x9)	Center for Integrated Earth System Information (CIESIN)
cod_bld_farea_202406 17_unpublished	2024	Building Area	Sum	Center for Integrated Earth System Information (CIESIN)
cod_buildings_count_ bcb_gl_100m_v1_1	2024	Building Count	Sum	Google
cod_buildings_count_ bcb_ms_100m_v1_1	2024	Building Count	Sum	Microsoft
cod_buildings_count_ pib_gl_100m_v1_1	2024	Building Count	Sum	Google
cod_buildings_count_ pib_ms_100m_v1_1	2024	Building Count	Sum	Microsoft
cod_buildings_cv_are a_bcb_gl_100m_v1_1	2024	Building Area	Sum	Google
cod_buildings_cv_are a_bcb_ms_100m_v1_1	2024	Building Area	Sum	Microsoft
cod_buildings_cv_are a_pib_gl_100m_v1_1	2024	Building Area	Sum	Google
cod_buildings_cv_are a_pib_ms_100m_v1_1	2024	Building Area	Sum	Microsoft
cod_buildings_cv_len gth_bcb_gl_100m_v1_1	2024	Building Perimeter	Sum	Google
cod_buildings_cv_len gth_bcb_ms_100m_v1_1	2024	Building Perimeter	Sum	Microsoft
cod_buildings_cv_len gth_pib_gl_100m_v1_1	2024	Building Perimeter	Sum	Google
cod_buildings_cv_len gth_pib_ms_100m_v1_1	2024	Building Perimeter	Sum	Microsoft
cod_buildings_densit y_bcb_gl_100m_v1_1	2024	Building Density	Sum	Google
cod_buildings_densit y_bcb_ms_100m_v1_1	2024	Building Density	Sum	Microsoft
cod_buildings_densit y_pib_gl_100m_v1_1	2024	Building Density	Sum	Google
cod_buildings_densit y_pib_ms_100m_v1_1	2024	Building Density	Sum	Microsoft
cod_buildings_mean_a rea_bcb_gl_100m_v1_1	2024	Building Area	Mean	Google
cod_buildings_mean_a rea_bcb_ms_100m_v1_1	2024	Building Area	Mean	Microsoft
cod_buildings_mean_a rea_pib_gl_100m_v1_1	2024	Building Area	Mean	Google
cod_buildings_mean_a rea_pib_ms_100m_v1_1	2024	Building Area	Mean	Microsoft
cod_buildings_mean_l ength_bcb_gl_100m_v1_1	2024	Building Perimeter	Mean	Google
cod_buildings_mean_l ength_bcb_ms_100m_v1_1	2024	Building Perimeter	Mean	Microsoft
cod_buildings_mean_l ength_pib_gl_100m_v1_1	2024	Building Perimeter	Mean	Google
cod_buildings_mean_l ength_pib_ms_100m_v1_1	2024	Building Perimeter	Mean	Microsoft
cod_buildings_total_ area_bcb_gl_100m_v1_ 1	2024	Building Area	Sum	Google
cod_buildings_total_ area_bcb_ms_100m_v1_ 1	2024	Building Area	Sum	Microsoft
cod_buildings_total_ area_pib_gl_100m_v1_ 1	2024	Building Area	Sum	Google
cod_buildings_total_ area_pib_ms_100m_v1_ 1	2024	Building Area	Sum	Microsoft

cod_buildings_total_ length_bcb_gl_100m_v 1_1	2024	Building Perimeter	Sum	Google
cod_buildings_total_ length_bcb_ms_100m_v 1_1	2024	Building Perimeter	Sum	Microsoft
cod_buildings_total_ length_pib_gl_100m_v 1_1	2024	Building Perimeter	Sum	Google
cod_buildings_total_ length_pib_ms_100m_v 1_1	2024	Building Perimeter	Sum	Microsoft
cod_built_binary_100 m_v1_2015	2015	Building Count	Presence/ Absence	European Commission Joint Research Centre (JRC)
cod_built_binary_100 m_v1_2020	2020	Building Count	Presence/ Absence	European Commission Joint Research Centre (JRC)
cod_built_binary_100 m_v1_2025	2025	Building Count	Presence/ Absence	European Commission Joint Research Centre (JRC)
cod_built_binary_100 m_v1_2030	2030	Building Count	Presence/ Absence	European Commission Joint Research Centre (JRC)
cod_built_binary_ghs_wfgw_100m_v1_2015	2015	Building Count	Presence/ Absence	European Commission Joint Research Centre (JRC)
cod_built_binary_ghs_wfgw_100m_v1_2020	2020	Building Count	Presence/ Absence	European Commission Joint Research Centre (JRC)
cod_built_binary_ghs_wfgw_100m_v1_2025	2025	Building Count	Presence/ Absence	European Commission Joint Research Centre (JRC)
cod_built_binary_ghs_wfgw_100m_v1_2030	2030	Building Count	Presence/ Absence	European Commission Joint Research Centre (JRC)
cod_built_s_100m_v1_ 2015	2015	Building Area	Sum	European Commission Joint Research Centre (JRC)
cod_built_s_100m_v1_ 2020	2020	Building Area	Sum	European Commission Joint Research Centre (JRC)
cod_built_s_100m_v1_ 2025	2025	Building Area	Sum	European Commission Joint Research Centre (JRC)
cod_built_s_100m_v1_ 2030	2030	Building Area	Sum	European Commission Joint Research Centre (JRC)
cod_built_s_ghs_wfgw_100m_v1_2015	2015	Building Area	Sum	European Commission Joint Research Centre (JRC)
cod_built_s_ghs_wfgw_100m_v1_2020	2020	Building Area	Sum	European Commission Joint Research Centre (JRC)
cod_built_s_ghs_wfgw_100m_v1_2025	2025	Building Area	Sum	European Commission Joint Research Centre (JRC)
cod_built_s_ghs_wfgw_100m_v1_2030	2030	Building Area	Sum	European Commission Joint Research Centre (JRC)
cod_built_s_nres_ghs_wosm_100m_v1_2015	2015	Building Area	Sum	European Commission Joint Research Centre (JRC)
cod_built_s_nres_ghs_wosm_100m_v1_2016	2016	Building Area	Sum	European Commission Joint Research Centre (JRC)
cod_built_s_nres_ghs_wosm_100m_v1_2017	2017	Building Area	Sum	European Commission Joint Research Centre (JRC)
cod_built_s_nres_ghs_wosm_100m_v1_2018	2018	Building Area	Sum	European Commission Joint Research Centre (JRC)
cod_built_s_nres_ghs_wosm_100m_v1_2019	2019	Building Area	Sum	European Commission Joint Research Centre (JRC)
cod_built_s_nres_ghs_wosm_100m_v1_2020	2020	Building Area	Sum	European Commission Joint Research Centre (JRC)
cod_built_s_nres_ghs_wosm_100m_v1_2021	2021	Building Area	Sum	European Commission Joint Research Centre (JRC)
cod_built_s_nres_ghs_wosm_100m_v1_2022	2022	Building Area	Sum	European Commission Joint Research Centre (JRC)

cod_built_s_nres_ghs_wosm_100m_v1_2023	2023	Building Area	Sum	European Commission Joint Research Centre (JRC)
cod_built_s_nres_ghs_wosm_100m_v1_2024	2024	Building Area	Sum	European Commission Joint Research Centre (JRC)
cod_built_s_nres_ghs_wosm_100m_v1_2025	2025	Building Area	Sum	European Commission Joint Research Centre (JRC)
cod_built_s_nres_ghs_wosm_100m_v1_2026	2026	Building Area	Sum	European Commission Joint Research Centre (JRC)
cod_built_s_nres_ghs_wosm_100m_v1_2027	2027	Building Area	Sum	European Commission Joint Research Centre (JRC)
cod_built_s_nres_ghs_wosm_100m_v1_2028	2028	Building Area	Sum	European Commission Joint Research Centre (JRC)
cod_built_s_nres_ghs_wosm_100m_v1_2029	2029	Building Area	Sum	European Commission Joint Research Centre (JRC)
cod_built_s_nres_ghs_wosm_100m_v1_2030	2030	Building Area	Sum	European Commission Joint Research Centre (JRC)
cod_built_v_100m_v1_ 2015	2015	Building Volume	Sum	European Commission Joint Research Centre (JRC)
cod_built_v_100m_v1_ 2020	2020	Building Volume	Sum	European Commission Joint Research Centre (JRC)
cod_built_v_100m_v1_ 2025	2025	Building Volume	Sum	European Commission Joint Research Centre (JRC)
cod_built_v_100m_v1_ 2030	2030	Building Volume	Sum	European Commission Joint Research Centre (JRC)
cod_built_v_ghs_wfgw_100m_v1_2015	2015	Building Volume	Sum	European Commission Joint Research Centre (JRC)
cod_built_v_ghs_wfgw_100m_v1_2020	2020	Building Volume	Sum	European Commission Joint Research Centre (JRC)
cod_built_v_ghs_wfgw_100m_v1_2025	2025	Building Volume	Sum	European Commission Joint Research Centre (JRC)
cod_built_v_ghs_wfgw_100m_v1_2030	2030	Building Volume	Sum	European Commission Joint Research Centre (JRC)
cod_built_v_nres_ghs_wosm_100m_v1_2015	2015	Building Volume	Sum	European Commission Joint Research Centre (JRC)
cod_built_v_nres_ghs_wosm_100m_v1_2016	2016	Building Volume	Sum	European Commission Joint Research Centre (JRC)
cod_built_v_nres_ghs_wosm_100m_v1_2017	2017	Building Volume	Sum	European Commission Joint Research Centre (JRC)
cod_built_v_nres_ghs_wosm_100m_v1_2018	2018	Building Volume	Sum	European Commission Joint Research Centre (JRC)
cod_built_v_nres_ghs_wosm_100m_v1_2019	2019	Building Volume	Sum	European Commission Joint Research Centre (JRC)
cod_built_v_nres_ghs_wosm_100m_v1_2020	2020	Building Volume	Sum	European Commission Joint Research Centre (JRC)
cod_built_v_nres_ghs_wosm_100m_v1_2021	2021	Building Volume	Sum	European Commission Joint Research Centre (JRC)
cod_built_v_nres_ghs_wosm_100m_v1_2022	2022	Building Volume	Sum	European Commission Joint Research Centre (JRC)
cod_built_v_nres_ghs_wosm_100m_v1_2023	2023	Building Volume	Sum	European Commission Joint Research Centre (JRC)
cod_built_v_nres_ghs_wosm_100m_v1_2024	2024	Building Volume	Sum	European Commission Joint Research Centre (JRC)
cod_built_v_nres_ghs_wosm_100m_v1_2025	2025	Building Volume	Sum	European Commission Joint Research Centre (JRC)
cod_built_v_nres_ghs_wosm_100m_v1_2026	2026	Building Volume	Sum	European Commission Joint Research Centre (JRC)

cod_built_v_nres_ghs_wosm_100m_v1_2027	2027	Building Volume	Sum	European Commission Joint Research Centre (JRC)
cod_built_v_nres_ghs_wosm_100m_v1_2028	2028	Building Volume	Sum	European Commission Joint Research Centre (JRC)
cod_built_v_nres_ghs_wosm_100m_v1_2029	2029	Building Volume	Sum	European Commission Joint Research Centre (JRC)
cod_built_v_nres_ghs_wosm_100m_v1_2030	2030	Building Volume	Sum	European Commission Joint Research Centre (JRC)
cod_c3s_lc_l4_lccs_2 020_11_100m_dist	2020	Cropland, Natural Vegetation Distance	Euclidean Distance	European Space Agency (ESA)
cod_c3s_lc_l4_lccs_2 020_130_100m_dist	2020	Shrubland Distance	Euclidean Distance	European Space Agency (ESA)
cod_c3s_lc_l4_lccs_2 020_140_100m_dist	2020	Herbaceous Cover, Grassland, Mosses Distance	Euclidean Distance	European Space Agency (ESA)
cod_c3s_lc_l4_lccs_2 020_150_100m_dist	2020	Sparse Vegetation Distance	Euclidean Distance	European Space Agency (ESA)
cod_c3s_lc_l4_lccs_2 020_160_100m_dist	2020	Herbaceous Cover, Flooded, Fresh/Saline/Brackish Water Distance	Euclidean Distance	European Space Agency (ESA)
cod_c3s_lc_l4_lccs_2 020_190_100m_dist	2020	Urban Areas Distance	Euclidean Distance	European Space Agency (ESA)
cod_c3s_lc_l4_lccs_2 020_200_100m_dist	2020	Bare Areas Distance	Euclidean Distance	European Space Agency (ESA)
cod_c3s_lc_l4_lccs_2 020_210_100m_dist	2020	Water Bodies, Permanent Snow Distance	Euclidean Distance	European Space Agency (ESA)
cod_c3s_lc_l4_lccs_2 020_40_100m_dist	2020	Tree Cover Distance	Euclidean Distance	European Space Agency (ESA)
cod_coastline_dst_10 0m_v1	2021	Coastline Distance	Euclidean Distance	European Space Agency (ESA)
cod_dist_inland_wate r_100m_esa_2021_v1	2021	Inland Water Distance	Euclidean Distance	European Space Agency (ESA)
cod_elevation_merit1 03_100m_v1	2000	Elevation	Mean	European Space Agency (ESA)
cod_esalc_11_dst_201 5_100m_v1	2015	Cropland, Natural Vegetation Distance	Euclidean Distance	European Space Agency (ESA)
cod_esalc_11_dst_201 6_100m_v1	2016	Cropland, Natural Vegetation Distance	Euclidean Distance	European Space Agency (ESA)
cod_esalc_11_dst_201 7_100m_v1	2017	Cropland, Natural Vegetation Distance	Euclidean Distance	European Space Agency (ESA)
cod_esalc_11_dst_201 8_100m_v1	2018	Cropland, Natural Vegetation Distance	Euclidean Distance	European Space Agency (ESA)
cod_esalc_11_dst_201 9_100m_v1	2019	Cropland, Natural Vegetation Distance	Euclidean Distance	European Space Agency (ESA)
cod_esalc_11_dst_202 0_100m_v1	2020	Cropland, Natural Vegetation Distance	Euclidean Distance	European Space Agency (ESA)
cod_esalc_11_dst_202 1_100m_v1	2021	Cropland, Natural Vegetation Distance	Euclidean Distance	European Space Agency (ESA)
cod_esalc_11_dst_202 2_100m_v1	2022	Cropland, Natural Vegetation Distance	Euclidean Distance	European Space Agency (ESA)
cod_esalc_130_dst_20 15_100m_v1	2015	Shrubland Distance	Euclidean Distance	European Space Agency (ESA)
cod_esalc_130_dst_20 16_100m_v1	2016	Shrubland Distance	Euclidean Distance	European Space Agency (ESA)

cod_esalc_130_dst_20 17_100m_v1	2017	Shrubland Distance	Euclidean Distance	European Space Agency (ESA)
cod_esalc_130_dst_20 18_100m_v1	2018	Shrubland Distance	Euclidean Distance	European Space Agency (ESA)
cod_esalc_130_dst_20 19_100m_v1	2019	Shrubland Distance	Euclidean Distance	European Space Agency (ESA)
cod_esalc_130_dst_20 20_100m_v1	2020	Shrubland Distance	Euclidean Distance	European Space Agency (ESA)
cod_esalc_130_dst_20 21_100m_v1	2021	Shrubland Distance	Euclidean Distance	European Space Agency (ESA)
cod_esalc_130_dst_20 22_100m_v1	2022	Shrubland Distance	Euclidean Distance	European Space Agency (ESA)
cod_esalc_140_dst_20 15_100m_v1	2015	Herbaceous Cover, Grassland, Mosses Distance	Euclidean Distance	European Space Agency (ESA)
cod_esalc_140_dst_20 16_100m_v1	2016	Herbaceous Cover, Grassland, Mosses Distance	Euclidean Distance	European Space Agency (ESA)
cod_esalc_140_dst_20 17_100m_v1	2017	Herbaceous Cover, Grassland, Mosses Distance	Euclidean Distance	European Space Agency (ESA)
cod_esalc_140_dst_20 18_100m_v1	2018	Herbaceous Cover, Grassland, Mosses Distance	Euclidean Distance	European Space Agency (ESA)
cod_esalc_140_dst_20 19_100m_v1	2019	Herbaceous Cover, Grassland, Mosses Distance	Euclidean Distance	European Space Agency (ESA)
cod_esalc_140_dst_20 20_100m_v1	2020	Herbaceous Cover, Grassland, Mosses Distance	Euclidean Distance	European Space Agency (ESA)
cod_esalc_140_dst_20 21_100m_v1	2021	Herbaceous Cover, Grassland, Mosses Distance	Euclidean Distance	European Space Agency (ESA)
cod_esalc_140_dst_20 22_100m_v1	2022	Herbaceous Cover, Grassland, Mosses Distance	Euclidean Distance	European Space Agency (ESA)
cod_esalc_150_dst_20 15_100m_v1	2015	Sparse Vegetation Distance	Euclidean Distance	European Space Agency (ESA)
cod_esalc_150_dst_20 16_100m_v1	2016	Sparse Vegetation Distance	Euclidean Distance	European Space Agency (ESA)
cod_esalc_150_dst_20 17_100m_v1	2017	Sparse Vegetation Distance	Euclidean Distance	European Space Agency (ESA)
cod_esalc_150_dst_20 18_100m_v1	2018	Sparse Vegetation Distance	Euclidean Distance	European Space Agency (ESA)
cod_esalc_150_dst_20 19_100m_v1	2019	Sparse Vegetation Distance	Euclidean Distance	European Space Agency (ESA)
cod_esalc_150_dst_20 20_100m_v1	2020	Sparse Vegetation Distance	Euclidean Distance	European Space Agency (ESA)
cod_esalc_150_dst_20 21_100m_v1	2021	Sparse Vegetation Distance	Euclidean Distance	European Space Agency (ESA)
cod_esalc_150_dst_20 22_100m_v1	2022	Sparse Vegetation Distance	Euclidean Distance	European Space Agency (ESA)
cod_esalc_160_dst_20 15_100m_v1	2015	Herbaceous Cover, Flooded, Fresh/Saline/Brackish Water Distance	Euclidean Distance	European Space Agency (ESA)

cod_esalc_160_dst_20 16_100m_v1	2016	Herbaceous Cover, Flooded, Fresh/Saline/Brackish Water Distance	Euclidean Distance	European Space Agency (ESA)
cod_esalc_160_dst_20 17_100m_v1	2017	Herbaceous Cover, Flooded, Fresh/Saline/Brackish Water Distance	Euclidean Distance	European Space Agency (ESA)
cod_esalc_160_dst_20 18_100m_v1	2018	Herbaceous Cover, Flooded, Fresh/Saline/Brackish Water Distance	Euclidean Distance	European Space Agency (ESA)
cod_esalc_160_dst_20 19_100m_v1	2019	Herbaceous Cover, Flooded, Fresh/Saline/Brackish Water Distance	Euclidean Distance	European Space Agency (ESA)
cod_esalc_160_dst_20 20_100m_v1	2020	Herbaceous Cover, Flooded, Fresh/Saline/Brackish Water Distance	Euclidean Distance	European Space Agency (ESA)
cod_esalc_160_dst_20 21_100m_v1	2021	Herbaceous Cover, Flooded, Fresh/Saline/Brackish Water Distance	Euclidean Distance	European Space Agency (ESA)
cod_esalc_160_dst_20 22_100m_v1	2022	Herbaceous Cover, Flooded, Fresh/Saline/Brackish Water Distance	Euclidean Distance	European Space Agency (ESA)
cod_esalc_190_dst_20 15_100m_v1	2015	Urban Areas Distance	Euclidean Distance	European Space Agency (ESA)
cod_esalc_190_dst_20 16_100m_v1	2016	Urban Areas Distance	Euclidean Distance	European Space Agency (ESA)
cod_esalc_190_dst_20 17_100m_v1	2017	Urban Areas Distance	Euclidean Distance	European Space Agency (ESA)
cod_esalc_190_dst_20 18_100m_v1	2018	Urban Areas Distance	Euclidean Distance	European Space Agency (ESA)
cod_esalc_190_dst_20 19_100m_v1	2019	Urban Areas Distance	Euclidean Distance	European Space Agency (ESA)
cod_esalc_190_dst_20 20_100m_v1	2020	Urban Areas Distance	Euclidean Distance	European Space Agency (ESA)
cod_esalc_190_dst_20 21_100m_v1	2021	Urban Areas Distance	Euclidean Distance	European Space Agency (ESA)
cod_esalc_190_dst_20 22_100m_v1	2022	Urban Areas Distance	Euclidean Distance	European Space Agency (ESA)
cod_esalc_200_dst_20 15_100m_v1	2015	Bare Areas Distance	Euclidean Distance	European Space Agency (ESA)
cod_esalc_200_dst_20 16_100m_v1	2016	Bare Areas Distance	Euclidean Distance	European Space Agency (ESA)
cod_esalc_200_dst_20 17_100m_v1	2017	Bare Areas Distance	Euclidean Distance	European Space Agency (ESA)
cod_esalc_200_dst_20 18_100m_v1	2018	Bare Areas Distance	Euclidean Distance	European Space Agency (ESA)
cod_esalc_200_dst_20 19_100m_v1	2019	Bare Areas Distance	Euclidean Distance	European Space Agency (ESA)
cod_esalc_200_dst_20 20_100m_v1	2020	Bare Areas Distance	Euclidean Distance	European Space Agency (ESA)
cod_esalc_200_dst_20 21_100m_v1	2021	Bare Areas Distance	Euclidean Distance	European Space Agency (ESA)

cod_esalc_200_dst_20 22_100m_v1	2022	Bare Areas Distance	Euclidean Distance	European Space Agency (ESA)
cod_esalc_210_dst_20 15_100m_v1	2015	Water Bodies, Permanent Snow Distance	Euclidean Distance	European Space Agency (ESA)
cod_esalc_210_dst_20 16_100m_v1	2016	Water Bodies, Permanent Snow Distance	Euclidean Distance	European Space Agency (ESA)
cod_esalc_210_dst_20 17_100m_v1	2017	Water Bodies, Permanent Snow Distance	Euclidean Distance	European Space Agency (ESA)
cod_esalc_210_dst_20 18_100m_v1	2018	Water Bodies, Permanent Snow Distance	Euclidean Distance	European Space Agency (ESA)
cod_esalc_210_dst_20 19_100m_v1	2019	Water Bodies, Permanent Snow Distance	Euclidean Distance	European Space Agency (ESA)
cod_esalc_210_dst_20 20_100m_v1	2020	Water Bodies, Permanent Snow Distance	Euclidean Distance	European Space Agency (ESA)
cod_esalc_210_dst_20 21_100m_v1	2021	Water Bodies, Permanent Snow Distance	Euclidean Distance	European Space Agency (ESA)
cod_esalc_210_dst_20 22_100m_v1	2022	Water Bodies, Permanent Snow Distance	Euclidean Distance	European Space Agency (ESA)
cod_esalc_40_dst_201 5_100m_v1	2015	Tree Cover Distance	Euclidean Distance	European Space Agency (ESA)
cod_esalc_40_dst_201 6_100m_v1	2016	Tree Cover Distance	Euclidean Distance	European Space Agency (ESA)
cod_esalc_40_dst_201 7_100m_v1	2017	Tree Cover Distance	Euclidean Distance	European Space Agency (ESA)
cod_esalc_40_dst_201 8_100m_v1	2018	Tree Cover Distance	Euclidean Distance	European Space Agency (ESA)
cod_esalc_40_dst_201 9_100m_v1	2019	Tree Cover Distance	Euclidean Distance	European Space Agency (ESA)
cod_esalc_40_dst_202 0_100m_v1	2020	Tree Cover Distance	Euclidean Distance	European Space Agency (ESA)
cod_esalc_40_dst_202 1_100m_v1	2021	Tree Cover Distance	Euclidean Distance	European Space Agency (ESA)
cod_esalc_40_dst_202 2_100m_v1	2022	Tree Cover Distance	Euclidean Distance	European Space Agency (ESA)
cod_ghs_built_s_e202 0_globe_r2023a_4326_ 3ss_v1_0_gl2_wfgw	2020	Building Area	Euclidean Distance	European Commission Joint Research Centre (JRC)
cod_ghs_built_s_nres_e2020_globe_r2023a_ 4326_3ss_v1_0_gl2	2020	Building Area	Euclidean Distance	European Commission Joint Research Centre (JRC)
cod_ghs_built_v_e202 0_globe_r2023a_4326_ 3ss_v1_0_gl2_wfgw	2020	Building Volume	Euclidean Distance	European Commission Joint Research Centre (JRC)
cod_ghs_built_v_nres_e2020_globe_r2023a_ 4326_3ss_v1_0_gl2	2020	Building Volume	Euclidean Distance	European Commission Joint Research Centre (JRC)
cod_ghs_built_v_with outnres_e2020_globe_ r2023a_4326_3ss_v1_0_gl2_wfgw	2020	Building Volume	Euclidean Distance	European Commission Joint Research Centre (JRC)
cod_highway_dist_osm_2023_100m_v1	2023	Highway Distance	Euclidean Distance	European Commission Joint Research Centre (JRC)
cod_inland_water_bin_100m_esa_2021_v1	2021	Inland Water Distance	Euclidean Distance	European Space Agency (ESA)

cod_inland_water_pc2 5_bin_100m_esa_2021_v1	2021	Inland Water Distance	Euclidean Distance	European Space Agency (ESA)
cod_inland_water_pc5 0_bin_100m_esa_2021_v1	2021	Inland Water Distance	Euclidean Distance	European Space Agency (ESA)
cod_inland_water_pc6 0_bin_100m_esa_2021_v1	2021	Inland Water Distance	Euclidean Distance	European Space Agency (ESA)
cod_inland_water_pc7 5_bin_100m_esa_2021_v1	2021	Inland Water Distance	Euclidean Distance	European Space Agency (ESA)
cod_inland_water_pc8 0_bin_100m_esa_2021_v1	2021	Inland Water Distance	Euclidean Distance	European Space Agency (ESA)
cod_inland_water_pc8 5_bin_100m_esa_2021_v1	2021	Inland Water Distance	Euclidean Distance	European Space Agency (ESA)
cod_inland_water_pc9 0_bin_100m_esa_2021_v1	2021	Inland Water Distance	Euclidean Distance	European Space Agency (ESA)
cod_inland_water_pct_100m_v1	2021	Inland Water Distance	Euclidean Distance	European Space Agency (ESA)
cod_ms_roads_binary_ 100m_v1	2023	Road Intersection	Presence/ Absence	Microsoft
cod_ms_roads_count_1 00m_v1	2023	Road Node Count	Sum	Microsoft
cod_ms_roads_density_100m_v1	2023	Road Density	Sum	Microsoft
cod_ms_roads_dst_100 m_v1	2023	Road Distance	Euclidean Distance	Microsoft
cod_ms_roads_total_l ength_100m_v1	2023	Roads Length	Sum	Microsoft
cod_ppt_2015_yravg_t c_100m_v1	2015	Precipitation	Mean	TerraClimate
cod_ppt_2016_yravg_t c_100m_v1	2016	Precipitation	Mean	TerraClimate
cod_ppt_2017_yravg_t c_100m_v1	2017	Precipitation	Mean	TerraClimate
cod_ppt_2018_yravg_t c_100m_v1	2018	Precipitation	Mean	TerraClimate
cod_ppt_2019_yravg_t c_100m_v1	2019	Precipitation	Mean	TerraClimate
cod_ppt_2020_yravg_t c_100m_v1	2020	Precipitation	Mean	TerraClimate
cod_ppt_2021_yravg_t c_100m_v1	2021	Precipitation	Mean	TerraClimate
cod_ppt_2022_yravg_t c_100m_v1	2022	Precipitation	Mean	TerraClimate
cod_rd_intrs_dist_osm_2023_100m_v1	2023	Road Intersection Distance	Euclidean Distance	OpenStreetMap
cod_slope_merit103_1 00m_v1	2000	Terrain Slope	None	European Space Agency (ESA)
cod_tavg_2015_tlst_1 00m_v1	2015	Land Surface Temperature	Mean	Moderate Resolution Imaging Spectroradiometer (MODIS)
cod_tavg_2016_tlst_1 00m_v1	2016	Land Surface Temperature	Mean	Moderate Resolution Imaging Spectroradiometer (MODIS)
cod_tavg_2017_tlst_1 00m_v1	2017	Land Surface Temperature	Mean	Moderate Resolution Imaging Spectroradiometer (MODIS)
cod_tavg_2018_tlst_1 00m_v1	2018	Land Surface Temperature	Mean	Moderate Resolution Imaging Spectroradiometer (MODIS)
cod_tavg_2019_tlst_1 00m_v1	2019	Land Surface Temperature	Mean	Moderate Resolution Imaging Spectroradiometer (MODIS)
cod_tavg_2020_tlst_1 00m_v1	2020	Land Surface Temperature	Mean	Moderate Resolution Imaging Spectroradiometer (MODIS)

cod_tavg_2021_tlst_1 00m_v1	2021	Land Surface Temperature	Mean	Moderate Resolution Imaging Spectroradiometer (MODIS)
cod_tavg_2022_tlst_1 00m_v1	2022	Land Surface Temperature	Mean	Moderate Resolution Imaging Spectroradiometer (MODIS)
cod_viirs_fvf_2015_1 00m_v1	2015	Visible Infrared Imaging Radiometer Suite (FVF)	None	National Aeronautics and Space Administration (NASA)
cod_viirs_fvf_2016_1 00m_v1	2016	Visible Infrared Imaging Radiometer Suite (FVF)	None	National Aeronautics and Space Administration (NASA)
cod_viirs_fvf_2017_1 00m_v1	2017	Visible Infrared Imaging Radiometer Suite (FVF)	None	National Aeronautics and Space Administration (NASA)
cod_viirs_fvf_2018_1 00m_v1	2018	Visible Infrared Imaging Radiometer Suite (FVF)	None	National Aeronautics and Space Administration (NASA)
cod_viirs_fvf_2019_1 00m_v1	2019	Visible Infrared Imaging Radiometer Suite (FVF)	None	National Aeronautics and Space Administration (NASA)
cod_viirs_fvf_2020_1 00m_v1	2020	Visible Infrared Imaging Radiometer Suite (FVF)	None	National Aeronautics and Space Administration (NASA)
cod_viirs_fvf_2021_1 00m_v1	2021	Visible Infrared Imaging Radiometer Suite (FVF)	None	National Aeronautics and Space Administration (NASA)
cod_viirs_fvf_2022_1 00m_v1	2022	Visible Infrared Imaging Radiometer Suite (FVF)	None	National Aeronautics and Space Administration (NASA)
cod_viirs_nvf_2015_1 00m_v1	2015	Visible Infrared Imaging Radiometer Suite (NVF)	None	National Aeronautics and Space Administration (NASA)
cod_viirs_nvf_2016_1 00m_v1	2016	Visible Infrared Imaging Radiometer Suite (NVF)	None	National Aeronautics and Space Administration (NASA)
cod_viirs_nvf_2017_1 00m_v1	2017	Visible Infrared Imaging Radiometer Suite (NVF)	None	National Aeronautics and Space Administration (NASA)
cod_viirs_nvf_2018_1 00m_v1	2018	Visible Infrared Imaging Radiometer Suite (NVF)	None	National Aeronautics and Space Administration (NASA)
cod_viirs_nvf_2019_1 00m_v1	2019	Visible Infrared Imaging Radiometer Suite (NVF)	None	National Aeronautics and Space Administration (NASA)
cod_viirs_nvf_2020_1 00m_v1	2020	Visible Infrared Imaging Radiometer Suite (NVF)	None	National Aeronautics and Space Administration (NASA)
cod_viirs_nvf_2021_1 00m_v1	2021	Visible Infrared Imaging Radiometer Suite (NVF)	None	National Aeronautics and Space Administration (NASA)
cod_viirs_nvf_2022_1 00m_v1	2022	Visible Infrared Imaging Radiometer Suite (NVF)	None	National Aeronautics and Space Administration (NASA)
cod_waterbodies_dist_osm_2023_100m_v1	2023	Water Bodies Distance	Euclidean Distance	OpenStreetMap
cod_waterbodies_dist_osm_gl2_v1	2023	Water Bodies Distance	Euclidean Distance	World Database of Protected Areas (WDPA)

cod_wdpa_dist_2022_c at1_100m	2022	Protected Areas Distance (Category 1)	Euclidean Distance	World Database of Protected Areas (WDPA)
cod_wdpa_pre2015_cat 0_dist_100m_v1	2015	Protected Areas Distance (Category 0)	Euclidean Distance	World Database of Protected Areas (WDPA)
cod_wdpa_pre2015_cat 1_dist_100m_v1	2015	Protected Areas Distance (Category 1)	Euclidean Distance	World Database of Protected Areas (WDPA)
cod_wdpa_pre2016_cat 0_dist_100m_v1	2016	Protected Areas Distance (Category 0)	Euclidean Distance	World Database of Protected Areas (WDPA)
cod_wdpa_pre2016_cat 1_dist_100m_v1	2016	Protected Areas Distance (Category 1)	Euclidean Distance	World Database of Protected Areas (WDPA)
cod_wdpa_pre2017_cat 0_dist_100m_v1	2017	Protected Areas Distance (Category 0)	Euclidean Distance	World Database of Protected Areas (WDPA)
cod_wdpa_pre2017_cat 1_dist_100m_v1	2017	Protected Areas Distance (Category 1)	Euclidean Distance	World Database of Protected Areas (WDPA)
cod_wdpa_pre2018_cat 0_dist_100m_v1	2018	Protected Areas Distance (Category 0)	Euclidean Distance	World Database of Protected Areas (WDPA)
cod_wdpa_pre2018_cat 1_dist_100m_v1	2018	Protected Areas Distance (Category 1)	Euclidean Distance	World Database of Protected Areas (WDPA)
cod_wdpa_pre2019_cat 0_dist_100m_v1	2019	Protected Areas Distance (Category 0)	Euclidean Distance	World Database of Protected Areas (WDPA)
cod_wdpa_pre2019_cat 1_dist_100m_v1	2019	Protected Areas Distance (Category 1)	Euclidean Distance	World Database of Protected Areas (WDPA)
cod_wdpa_pre2020_cat 0_dist_100m_v1	2020	Protected Areas Distance (Category 0)	Euclidean Distance	World Database of Protected Areas (WDPA)
cod_wdpa_pre2020_cat 1_dist_100m_v1	2020	Protected Areas Distance (Category 1)	Euclidean Distance	World Database of Protected Areas (WDPA)
cod_wdpa_pre2021_cat 0_dist_100m_v1	2021	Protected Areas Distance (Category 0)	Euclidean Distance	World Database of Protected Areas (WDPA)
cod_wdpa_pre2021_cat 1_dist_100m_v1	2021	Protected Areas Distance (Category 1)	Euclidean Distance	World Database of Protected Areas (WDPA)
cod_wdpa_pre2022_cat 0_dist_100m_v1	2022	Protected Areas Distance (Category 0)	Euclidean Distance	World Database of Protected Areas (WDPA)
cod_wdpa_pre2022_cat 1_dist_100m_v1	2022	Protected Areas Distance (Category 1)	Euclidean Distance	World Database of Protected Areas (WDPA)
cod_wsf_evln_2005_20 15_v1_binary_0_nd_10 0m	2005-2015	Settlement Footprint	Presence/ Absence	World Settlement Footprint (FSF)
drc_access_to_cities_2015	2015	Access to Cities	Euclidean Distance	Weiss et al. 2018
drc_cop_2mtemp_2022_ mean_v3	2022	Temperature	Mean	Terra MODIS LST
drc_cop_2mtemp_2022_ sd	2022	Temperature	Standard Deviation	Terra MODIS LST
drc_cop_burntarea_20 21_mean_v3	2021	Burnt Area	Mean	Copernicus
drc_cop_burntarea_20 21_sd_v3	2021	Burnt Area	Standard Deviation	Copernicus
drc_cop_cloudcover_2 021_mean_v3	2021	Cloud Cover	Mean	Copernicus
drc_cop_cloudcover_2 021_sd_v3	2021	Cloud Cover	Standard Deviation	Copernicus
drc_cop_drymatterpro ductivity_2022_mean_ v3	2022	Dry Matter Productivity	Mean	Copernicus
drc_cop_drymatterpro ductivity_2022_sd	2022	Dry Matter Productivity	Standard Deviation	Copernicus
drc_cop_precip_2022_ mean_v3	2022	Precipitation	Mean	Copernicus
drc_cop_precip_2022_ sd	2022	Precipitation	Standard Deviation	Copernicus

drc_dst_acled_all_co nflict_2022_v3	2022	All Conflicts Location Distance	Euclidean Distance	Copernicus
drc_dst_acled_battle s_2022_v3	2022	Battles Location Distance	Euclidean Distance	Armed Conflict Location and Event Data (ACLED)
drc_dst_acled_explos ions_rv_2022_v3	2022	Explosions Location Distance	Euclidean Distance	Armed Conflict Location and Event Data (ACLED)
drc_dst_acled_protes ts_2022_v3	2022	Protests Location Distance	Euclidean Distance	Armed Conflict Location and Event Data (ACLED)
drc_dst_acled_riots_ 2022_v3	2022	Riots Location Distance	Euclidean Distance	Armed Conflict Location and Event Data (ACLED)
drc_dst_acled_strate gic_developments_202 2_v3	2022	Strategic Development Distance	Euclidean Distance	Armed Conflict Location and Event Data (ACLED)
drc_dst_acled_violen ce_against_civilians_2022_v3	2022	Violence Agains Civilians Distance	Euclidean Distance	Armed Conflict Location and Event Data (ACLED)
drc_dst_coastlines_2 022_v3	2022	Coastline Distance	Euclidean Distance	OpenStreetMap
drc_dst_osm_educatio nf_2023_v3	2023	Education Facilities Distance	Euclidean Distance	OpenStreetMap
drc_dst_osm_localroa ds_2023_v3	2023	Local Roads Distance	Euclidean Distance	OpenStreetMap
drc_dst_osm_mainroad s_2023_v3	2023	Main Roads Distance	Euclidean Distance	OpenStreetMap
drc_dst_osm_placesof worship_2023_v3	2023	Places of Worship Distance	Euclidean Distance	OpenStreetMap
drc_dst_osm_railways_2023_v3	2023	Railways Distance	Euclidean Distance	OpenStreetMap
drc_elevation_2000	2020	Elevation	Euclidean Distance	European Space Agency (ESA)
drc_ghs_residential_ 2020	2020	Residential Area	Euclidean Distance	European Commission Joint Research Centre (JRC)
drc_ndvi_avg_2021	2021	Normalized Difference Vegetation Index	Mean	Copernicus
drc_ndvi_std_2021	2021	Normalized Difference Vegetation Index	Standard Deviation	Copernicus
drc_slope_2000	2020	Terrain Slope	None	European Space Agency (ESA)
drc_viirs_2021	2021	Visible Infrared Imaging Radiometer Suite	None	National Aeronautics and Space Administration (NASA)
drc_walking_travelti me_hf	2020	Walking Travel Time	None	Malaria Atlas Project (MAP)
ghs_smod_e2030_globe_r2023a_54009_1000_v 2_0	2023	Settlement Class	Mode	European Commission Joint Research Centre (JRC)
grid3_cod_health_fac ilities_v1_0_all_euc dist	2024	All Health Facilities Distance	Euclidean Distance	Geo-Referenced Infrastructure and Demographic Data for Development (GRID3)
grid3_cod_health_fac ilities_v1_0_hospita l_eucdist	2024	Hospitals Distance	Euclidean Distance	Geo-Referenced Infrastructure and Demographic Data for Development (GRID3)
grid3_cod_health_fac ilities_v1_0_subset_ eucdist	2024	Health Facilities Distance	Euclidean Distance	Geo-Referenced Infrastructure and Demographic Data for Development (GRID3)

grid3_cod_roads_v1_0_all_eucdist	2024	Roads Distance	Euclidean Distance	Geo-Referenced Infrastructure and Demographic Data for Development (GRID3)
grid3_cod_stlext_gri d3_v02_urbcentre_euc dist	2024	Urban Center Distance	Euclidean Distance	Center for Integrated Earth System Information (CIESIN)
grid3_cod_stlext_gri d3_v02_urbcentre_euc dist_negativewithin	2024	Urban Center Distance	Euclidean Distance	Center for Integrated Earth System Information (CIESIN)
grid3_cod_stlext_gri d3_v02_urbcentre_urb cluster_eucdist	2024	Urban Center Distance	Euclidean Distance	Center for Integrated Earth System Information (CIESIN)
grid3_cod_stlext_gri d3_v02_urbcentre_urb cluster_eucdist_nega tivewithin	2024	Urban Center Distance	Euclidean Distance	Center for Integrated Earth System Information (CIESIN)
