# Supplementary material for: Tackling public health data gaps through Bayesian high-resolution population estimation: A case study of Kasaï-Oriental, Democratic Republic of the Congo
Source: PLOS Glob Public Health. 2025 Sep 4;5(9):e0005072. doi: 10.1371/journal.pgph.0005072 (PMC12410768; doi:10.1371/journal.pgph.0005072)
Supplement: Attachment — Submitted filename: Review.pdf [file pgph.0005072.s002.pdf]

Review for "Tackling public health data gaps through Bayesian high-resolution population estimation: a case study of Kasaï-Oriental, Democratic Republic of the Congo"

The manuscript focuses on creating and using a Bayesian hierarchical model to estimate population counts by levering a building count model and a population count model. This work is part of the greater goal to provide reliable population size estimates to organizations who require size estimates to implement health improvement measures.

I enjoyed reading this manuscript and recognize the importance of population size estimation in the context of improving public health. While I have no concerns about the scope and motivation behind this work, I have several comments regarding the statistical modeling sections, which I enumerate below.

- On Page 6, the authors state, "This approach leverages the flexibility of the hierarchical modelling framework to incorporate observational data and reflect model uncertainty through Bayesian credible intervals (CIs). Model uncertainty stems from limitations in the input data, including possible observational error in building and population counts and spatial aggregation issues in the covariates."

  While I agree with these sentences, this manuscript does not account for any observational error or spatial aggregation issues. I would recommend moving this statement to the discussion and clarify that if available, the proposed model can be adapted to account for these issues.

- The use of $\hat{B}_i$ as the mean of the Poisson confused me. Traditionally, we would write $B_i \sim Poisson(\mu_i)$, so that given the posterior distribution of μ_i, we can summarize the point estimate as $\hat{\mu_i}$ (where I use μ_i here to avoid the double use of B_i). As written in the manuscript, it initially reads as though there is no randomness in $\hat{B}_i$, until a later prior is placed on $\hat{B}_i$ in equation (2). This same confusing notation continues in the "Population count model" section.

- $\beta_s^{(B)}$ is not actually a matrix of random effects, as claimed by the authors, although this isn't clear until equation (9). The prior $\beta_s^{(B)} \sim N(0, 1)$ assumes $\beta_s^{(B)}$ is a fixed effect. I believe what the authors intended to say is that $\beta_s^{(B)}$ is a matrix of slopes between the interaction of the covariates in $X_i^{(B)}$ and the settlement class s. If $\beta_s^{(B)}$ were indeed random, it would have a prior of the form $\beta_s^{(B)} \sim N(0, \sigma^2)$, where σ^2 is estimated from the data.

- I would appreciate a little more discussion around the choice of informative priors. Why did the authors choose to have a fairly strong prior with a variance of 1, rather than a weaker prior with a larger variance? I would be interested in seeing how sensitive the results are to this choice of prior.

- The use of R^2 as a residual diagnostic is non-standard for a Bayesian model. Under the Bayesian paradigm, there would be a posterior distribution of R^2 values. I believe the R^2 in the manuscript was calculated using posterior means, and if used, should be clarified in the text (although Bayesian analogues exist).
