# Supplementary material for: Tackling public health data gaps through Bayesian high-resolution population estimation: A case study of Kasaï-Oriental, Democratic Republic of the Congo
Source: PLOS Glob Public Health. 2025 Sep 4;5(9):e0005072. doi: 10.1371/journal.pgph.0005072 (PMC12410768; doi:10.1371/journal.pgph.0005072)
Supplement: Attachment — Submitted filename: manuscript_review_1_response.pdf [file pgph.0005072.s003.pdf]

Gianluca Boo
School of Geography and Environmental Science
University of Southampton, Southampton, UK
gianluca.boo@soton.ac.uk

29/04/2025

Manuscript Revision

Dear Dr Wheldon,

Thank you for the opportunity to submit a revised version of our manuscript PGPH-D-24-02971, entitled "Tackling Public Health Data Gaps through Bayesian High-Resolution Population Estimation: A Case Study of Kasaï-Oriental, Democratic Republic of the Congo." We appreciate the time and effort that you and the reviewers have invested in providing feedback on our work, and we are grateful for the insightful comments and valuable suggestions that have helped improve our paper. We have addressed your comments, as well as those from the reviewers, and the changes are highlighted within the manuscript. Please find below our point-by-point response to the reviewers' comments and concerns.

Editor Comments:

Reviewer 3 noted that the link at the start of the manuscript pdf file leads to a dead site. Please make sure that the source data are publicly available, or explain why this isn't possible.

Thank you for highlighting this issue. We have now updated the link to the Zenodo repository (https://doi.org/10.5281/zenodo.14193338). The repository contains the input data and scripts used to develop the high-resolution population estimates, as well as the grid-cell posterior distributions and scripts to aggregate them within user-defined geographic boundaries.

Reviewer #1:

I found the topic of the paper to be interesting and the goal of joint modelling with rigorous assessment of uncertainty to be good. However, I have some issues with the presentation and I feel that it needs to become more clear.

Thank you for taking the time to thoroughly review our manuscript and for providing insightful comments and constructive criticism. We appreciate your positive assessment of our research focus on joint modelling and rigorous uncertainty assessment. We have carefully addressed each of your comments below, with specific references to the revisions made in the manuscript. We trust that the changes have improved the clarity and overall quality of the presentation.

1. Abstract: "The model exhibits limited bias, inaccuracy and imprecision..." This is a imprecise statement since it is subject to subjective assessment of what "limited" means. The authors should be more precise so that the readers of the abstract has an idea about the amount of bias and uncertainty.

Thank you for pointing out that the term "limited" could be interpreted subjectively. We have revised the abstract to remove this wording and instead provide precise numerical measures of bias, inaccuracy, and imprecision, reported in percentage terms.

2. Lines 87--89: This mitigation was very unclear to me. What does it mean to extend the "modeling environment". Are the authors calculating the population of a larger geographical area than intended? Clarification is needed.

We have revised and expanded the sentence to better explain our approach to addressing boundary misalignment. As the DRC government has not yet released official boundaries for the 26 provinces created in 2015, various unofficial administrative boundaries—often spatially misaligned—are currently in use. To avoid gaps arising from these discrepancies, we extended the modelling environment by applying a four-kilometre buffer to the OpenStreetMap boundaries. This ensures complete coverage and accounts for potential boundary misalignments.

3. Lines 92--109: Not all readers will be familiar with the micro-census data. I would appreciate more information. How were the micro-census clusters selected? Are they of uniform size?

We have expanded the relevant paragraph to describe the sampling frame and sampling design in greater detail, including how micro-census clusters were selected and whether they are of uniform size. In particular, we reference gridEZ—an algorithm that generates enumeration zones (EZs) based on user-defined target population and geographic size. Additional details on the methods, sampling frame, and micro-census survey data can be found in Boo et al. (2021).

4. Lines 111--138: There is too little information about the 292 datasets. Why exactly 292 datasets? Were transformations applied to the 292 datasets? Why were they reduced to exactly 5 datasets? What was the criteria for inclusion/non-inclusion? How large are the micro-clusters compared to the approximately 100 m x 100 m grid cells?

We have substantially expanded this section to describe their origins, processing, and transformations, as well as the covariate selection process. The datasets were sourced from various open-access repositories available at the time of this study, and a complete list is now included in the Appendix. Because multiple datasets often represented the same underlying feature but were provided by different sources, they differed in spatial properties, summary statistics, and temporal coverage. Using Pearson correlation tests, we identified which datasets were significantly correlated ($p<0.05$) with log-population densities and log-building counts. Among those, many were also significantly correlated ($p<0.05$) with each other, which would introduce redundancy. Consequently, we selected five datasets that offered complementary information for our model. We discuss these findings and the broader need for alternative spatial datasets in data-scarce contexts in the second paragraph of the Discussion section.

5. Line 135: Minor comment. I would replace "the mean" with "the average" and "standard deviation" with "empirical standard deviation" (since these are just summary quantities for the rasters)

Thank you for this suggestion. We have replaced “mean” with “average” and “standard deviation” with “empirical standard deviation” throughout the manuscript to more accurately reflect that these are summary quantities for the rasters.

6. Lines 140--: The statistical model is confusing to me. A data model is described for observed clusters, but how does it extend to unobserved grid cells? It would be very beneficial to first formulate the relationship between mean and covariates at the grid cell level (so that it is defined for all grid cells), and then describe how the micro-census data informs about the grid cell means.

Thank you for highlighting this point. We have thoroughly restructured the statistical model section to address both this comment and the subsequent ones. In particular, we now clearly describe how the

model transitions from micro-census cluster-level observations to grid-cell level predictions, and finally to areal-level aggregations, ensuring a more coherent and transparent presentation. Following the feedback of Reviewer #3, we have adopted weakly informative priors and re-run the entire analysis. The results presented in the revised version of this manuscript are slightly different.

7. Lines 140--: The use of the word Poisson process needs more description. It was very confusing to me. The index i indexes micro clusters so are the authors claiming that this is a Poisson process on micro clusters? Maybe what the authors intended is that B_i (where i indexes cells) is a discretized Poisson process where the intensity is given by log(\mu_i) = covariates+random effect? Then each microclusters j = 1,..., 213 corresponds to cells i[j] (???) which is then observed exactly.

Thank you for pointing out the confusion regarding the term "Poisson process." We have clarified how the Poisson distribution is used both at the micro-census-cluster level and at the grid-cell level. Specifically, we explain that B_i(indexed by i) represents a Poisson process with intensity given by $\lambda_i^{(B)}$,which is drawn from a Log Normal distribution with mean $\mu_i^{(B)}$arising from a linear regression. We also clarify how parameters estimated at the micro-census-cluster level are passed at the grid-cell level using respective settlement class allocation.

8. Lines 140--: There is a need to write something like s[i] to indicate that settlement class is determined by the grid cell.

Thank you for rising this point. We understand that our original model description may have contributed to a misunderstanding of the role of the settlement class, and we have since revised it to clarify its function. In our revised version, parameters like $\sigma_s^{(B)}$and $\alpha_s^{(B)}$are not indexed because they are shared across all clusters in class s. However, we clarified that this is linked to the location of the individual clusters and the grid cells.

9. Lines 151: It is claimed that \hat{B}_i is on logarithmic scale, but this cannot be true?

Thank you for noticing this oversight. We have removed any reference $\hat{B}_i$ being on a logarithmic scale to ensure accuracy in the description.

10. Line 157: It might be better to write LogNormal than log\mathcal{N} since the latter could be confused with the logarithm of a normal distributed variable (which does not make sense).

We have replaced log\mathcal{N} with LogNormal throughout the manuscript to avoid any potential confusion.

11. Lines 164--178: The same comments are valid here as in points 6.--10.

We have considered point 6 to 10 above in the original "Population count model" section—now "Population density" section.

12. Line 168: The "." should be a "\cdot".

We have replaced "." with " ·" in the equations to ensure proper mathematical notation.

13: Line 177: I don't understand the discussion about "random effects". Isn't the description in Equation (5) implicitly the inclusion of a random effect in the log-linear model?

Thank you for your comment. To clarify, while we included random effects in the building counts model, we used fixed effects in the population density model. We have revised the discussion about random effect to make this distinction explicit.

14. Lines 140--: NB. The authors present a Bayesian model. Then there is a need to be careful with the conditioning in the equations. E.g., Equation (1) is wrong. It is B_i | \hat{B}_i which is Poisson(\hat{B}_i). The same is true in Equation (2), you need to condition on \mu_i^(B) and \sigma_s^(B) for this to be logNormal. Please check all the equations.

We agree with the reviewer. We have carefully checked all equations to ensure proper conditioning throughout the manuscript.

15. Line 192: "5000 iterations" and "500 warm-up iterations" are not useful in it itself without knowing anything about the mixing properties of the Markov chain. Could the authors provide some metric like effective sample size to describe how informative 5000 iterations are?

We have included an assessment of Bulk Effective Sample Size in the first paragraph of the Results section. The model estimated a Bulk Effective Sample Size of 2,271, indicating that the posterior quantile estimates are reliable.

16. Lines 207--208: This sentence was unclear to me since the model is explicitly defined on the micro-census clusters. How can it be extended? If you describe the model more clearly, I believe this problem will go away. But I would be interested in a clarification about whether the "random effect"/logNormal is used also for unobserved grid cells?

We have thoroughly revised this section to provide a clearer description of the data model, explicitly detailing how it extends from micro-census-cluster-level observations to grid-cell-level predictions.

17. Line 212: Minor comment. I would write "... conditioned on the observed data under the model assumptions". It is a bit imprecise to state that you condition on the model assumptions.

We have revised this sentence to improve precision, ensuring that it correctly reflects conditioning on the observed data under the model assumptions.

18. Line 217: It would be nice for the reader to elaborate on what is meant by "comparable" measure.

The measure of uncertainty—width of the 95% credible interval relative to the mean—is a standardized way to assess uncertainty, making it more comparable across different models, datasets, or contexts. We replaced the term "comparable" with "standardised" to reflect the nature of the measurement more accurately.

19. Lines 295--297: This was confusing to me. You only fit the joint model, right? Then what does it mean that "the population count model does not noticeably improve the ability to capture variability in the observed data"?

We have rephrased this sentence focussing on the interpretation of the R2 value and how it relates to the contribution of the population count model in capturing variability in the observed data.

20. Table 2: Why is in-sample properties interesting? The model includes a random effect so in-sample properties might be very different from out-of-sample properties?

In Table 2, we present in-sample rather than out-of-sample properties because they reflect the goodness-of-fit for the micro-census-cluster-level model. These fitted parameters are then used for

grid-cell-level predictions, making the in-sample evaluation relevant to understanding model performance at the level where it is trained.

21. Lines 313--314: How were different age and sex classes suddenly introduced? There is nothing in the described model about this. The authors need more details here.

As part of this work, we estimated age and sex distributions by applying demographic pyramid proportions to the mean population counts. However, since these estimates are not directly discussed in the manuscript, we have removed all references to them to avoid potential confusion.

22. Line 359: I find the statement "novel Bayesian model" to vague. Novel in which sense?

We have clarified this statement by specifying that the novelty lies in the joint estimation of building and population counts at high spatial resolution.

23. Discussion: A concern for me would be that there is an assumption that the "residuals" from the mean in each grid cell are iid. I would believe there is considerable spatial structure in this residuals. The consequence of ignoring this would typically be to underestimate uncertainty in the aggregate estimates (for larger areas). I think this should be discussed. There is no out-of-sample evalutions to assess this potential issue either.

We have expanded the final paragraph of the “Model Fit” section to include a thorough assessment of spatial structure in the model residuals. Specifically, we conducted local and global Moran’s I tests on the residuals across different distance classes. The results indicate significant ($p<0.05$) but limited spatial autocorrelation ($I = 0.15$) only for the smallest distance class (0.05). The analysis of local Moran’s I patterns revealed that spatial autocorrelation is driven by systematic underpredictions in three remote clusters in the western part of the province. Given the small magnitude of this effect, we argue that the assumption of iid residuals remains reasonable. We added a sentence at the beginning of the second paragraph of the Discussion section to clarify this point.

Reviewer #2:

1. The paper addresses an important topic and a real challenge in low resource settings. Thank you for working on this.
2. I am not a statistician, and I cannot comment on the quality and novelty of the model / approach, nor on the extent to which it is correct. I would therefore defer to a statistician for this appraisal.
3. I accepted to review the paper because I was expecting more implications for public health, given that the journal is Plos Global Health. I would suggest clarifying and discussing points such as:

We are grateful for your recognition of the importance of this topic and its relevance to low-resource settings. We understand your position regarding the statistical aspects of the paper and appreciate your honest perspective. We acknowledge your expectations regarding public health implications and have clarified and expanded our discussion accordingly.

i) The complexity to estimate population for health areas/ zones given that they differ from the DRC administrative divisions.

We appreciate the reviewer for highlighting this important point. Estimating populations for health areas and zones, which do not align with the DRC’s administrative divisions, remains a persistent challenge. In our study, we address this issue by leveraging a gridded framework, which effectively resolves spatial

misalignment between administrative and operational health boundaries. To clarify this, we have added a sentence discussing the benefits of the gridded framework in the fourth paragraph of the Discussion section. Additionally, we have referenced Leyk et al. (2019), who provide an extensive discussion on how gridded population estimates offer a flexible and adaptable approach for estimating population distributions across various use cases.

ii) The feasibility for national authorities to learn from / replicate / update this approach for policy making, and the related resources and skillset needed.

We appreciate the reviewer's valuable comment. The feasibility of national authorities adopting, replicating, and updating this approach is indeed crucial for policymaking. To address this key challange, we have thoroughly revised the last paragraph of the Discussion section to emphasize that while the level of effort required is largely context-dependent, recent initiatives have demonstrated notable successes. For instance, the Colombian National Administrative Department of Statistics has successfully generated Bayesian population estimates for regions that are difficult to access. Additionally, we have expanded our discussion to stress that Bayesian high-resolution population estimates should not only be technically accessible but also relevant and user-friendly. Ensuring their applicability to public health data gaps is essential for improving planning accuracy and supporting effective interventions.

iii) The feasibility, cost, and duration related to the implementation of the micro-census.

Thank you for your insightful comment. We have added a third paragraph in the Discussion section elaborating on the feasibility, cost, and duration of implementing the micro-census. Specifically, we note that the survey was completed in approximately two months, at a significantly lower cost compared to a full enumeration. However, we are unable to disclose specific financial details of the micro-census. Additionally, we acknowledge the logistical challenges posed by insecurity and natural hazards, which highlight the difficulties of conducting a full enumeration in larger or hard-to-reach areas.

iv) The need for and the challenges related to updating the population estimates and include population displacement (should the region be affected again by insecurity, or should the model be replicated in other provinces of the DRC).

We appreciate the reviewer's concern regarding the need to update population estimates and account for population displacement, particularly in the face of ongoing insecurity or when replicating this model in other provinces of the DRC. This indeed poses a significant challenge. In the revised third paragraph of the Discussion section, we note that due to the logistical constraints encountered during the micro-census survey, it is unlikely that a similar micro-census could be replicated in the near future. However, we also highlight existing studies showing the potential of integrating alternative data sources—such as routinely collected health intervention campaign data and digital traces—to capture population dynamics in unsurveyed provinces. These approaches may offer a more sustainable method for updating population estimates in complex settings.

4. Furthermore, I would recommend clarifying why you chose to present the results on Kasai-Orientale, given that, if I understand correctly, several estimates have been produced with/ through the collaboration with GRID3 (https://grid3.org/geospatial-data-drc). It would be helpful to clarify whether the Kasai-Oriental approach is different in some ways and what the link with GRID3 is. You do mention GRID3 in the financial disclosure, but I would suggest explaining it in the paper itself.

We appreciate the reviewer's comment and have addressed this point by clarifying the rationale for focusing on Kasaï-Oriental. Specifically, we have updated the last sentence of the Introduction to state that this work was developed in support of an immunization campaign coordinated by GRID3 in the province. Additionally, we note that gridded population estimates have been produced in other parts of the DRC, with statistical models tailored to the specific data availability in each province.

5. While the lack of population estimates is big problem, it is not the only challenge to health service delivery in low-resource settings. I would recommend nuancing the introduction to recognize that lack of resources, limited access to population or health facilities, insecurity, logistical constraints, etc are major challenges that would persist even with reliable population estimates.

Thank you for this valuable suggestion. We have revised the first sentence of the Introduction to acknowledge the multiple challenges that impact the effectiveness of public health programs in low- and middle-income settings. In addition to the lack of reliable population estimates, we now recognize other critical barriers, such as resource constraints, limited access to populations and health facilities, insecurity, and logistical challenges, which continue to affect health service delivery.

Reviewer #3:

The manuscript focuses on creating and using a Bayesian hierarchical model to estimate population counts by levering a building count model and a population count model. This work is part of the greater goal to provide reliable population size estimates to organizations who require size estimates to implement health improvement measures.

I enjoyed reading this manuscript and recognize the importance of population size estimation in the context of improving public health. While I have no concerns about the scope and motivation behind this work, I have several comments regarding the statistical modeling sections, which I enumerate below.

Thank you for taking the time to review our manuscript and for your thoughtful comments. We greatly appreciate your insights and your recognition of the importance of population size estimation in the context of public health improvement. We have carefully considered your feedback and have addressed each of your comments in detail below. In particular, we have made extensive revisions to the statistical modelling sections to improve clarity and rigor, as outlined in the manuscript. Specific changes and justifications are provided in response to each point.

• On Page 6, the authors state, "This approach leverages the flexibility of the hierarchical modelling framework to incorporate observational data and reflect model uncertainty through Bayesian credible intervals (CIs). Model uncertainty stems from limitations in the input data, including possible observational error in building and population counts and spatial aggregation issues in the covariates." While I agree with these sentences, this manuscript does not account for any observational error or spatial aggregation issues. I would recommend moving this statement to the discussion and clarify that if available, the proposed model can be adapted to account for these issues.

We appreciate the reviewer's insight. We acknowledge that the manuscript does not explicitly account for observational error or spatial aggregation issues. For the sake of clarity, we have removed this statement from the text.

• The use of Bi as the mean of the Poisson confused me. Traditionally, we would write Bi ~ Poisson(μi), so that given the posterior distribution of μi, we can summarize the point estimate as μi (where I use μi here to avoid the double use of Bi). As written in the manuscript, it initially reads as though there is no randomness in Bi, until a later prior is placed on Bi in equation (2). This same confusing notation continues in the "Population count model" section.

Thank you for your feedback. We have revised the notation to clarify the distinction between the mean parameter and the random variable in the Poisson distribution. Specifically, we now use $\lambda_i^{(B)}$ as the mean of the Poisson distribution instead of $\hat{B}_i$ to avoid confusion. Additionally, we have ensured consistency in notation throughout the manuscript, including the Population count (now Population density) section.

• β(B)s is not actually a matrix of random effects, as claimed by the authors, although this isn't clear until equation (9). The prior β(B) s ~ N(0,1) assumes β(B) s is a fixed effect. I believe what the authors intended to say is that β(B) s is a matrix of slopes between the interaction of the covariates in X(B) i and the settlement class s. If β(B)s were indeed random, it would have a prior of the form β(B) s ~ N(0,σ2), where σ2 is estimated from the data.

We apologize for the confusion. Indeed, β(B)s is a matrix of random effects by settlement type, and reporting the prior on it was an oversight on our part. α(B)s remains a matrix of settlement-class-specific intercepts. We have corrected this in the revised manuscript, which now includes a thorough revision of the model description. In particular, following Reviewer #1's feedback, we provide more detail on how the model transitions from micro-census cluster-level observations to grid-cell-level predictions, and ultimately to areal-level aggregations, clarifying the roles of the different model parameters

• I would appreciate a little more discussion around the choice of informative priors. Why did the authors choose to have a fairly strong prior with a variance of 1, rather than a weaker prior with a larger variance? I would be interested in seeing how sensitive the results are to this choice of prior.

In our model, we initially specified informative priors for $\alpha_s^{(B)}$ and $\sigma_s^{(B)}$ using a reduced variance (i.e. 1) because we assumed that $X_i^{(B)}$ (the building count from settlement data) would require minimal correction. In our model, $\alpha_s^{(B)}$ accounts for settlement-class-specific additive bias, while $\sigma_s^{(B)}$ captures the residual variation specific to each settlement class. Similarly, for equation (9), we used comparable informative priors for $\alpha_s^{(D)}$and $\sigma_s^{(D)}$, based on the expectation of limited variability in a relatively homogeneous, sparsely populated setting. After receiving the reviewer's comment, we conducted extensive sensitivity tests using weaker priors with a larger variance (i.e. 10) and compared the resulting posterior density plots and estimates. Although the overall density distributions and posterior estimates remained similar, we observed more pronounced differences in the grid cell predictions. We attribute this to the long-tailed characteristics of the Log Normal distribution. Consequently, we decided to adopt less informative priors in the model and re-run the analysis. The updated model, along with replication instructions, has been uploaded to Zenodo (https://zenodo.org/records/15113660).

• The use of R2 as a residual diagnostic is non-standard for a Bayesian model. Under the Bayesian paradigm, there would be a posterior distribution of R2 values. I believe the R2 in the manuscript was calculated using posterior means, and if used, should be clarified in the text (although Bayesian analogues exist).

Thank you for this insightful comment. We acknowledge that R2 is not a standard residual diagnostic under the Bayesian paradigm. Indeed, we calculated R2 using the posterior means. We chose to include this metric because it is a widely recognized measure of model fit, familiar to audiences beyond the Bayesian community. To address your concern, we have clarified in the last paragraph of the Statistical model section that the R2 calculation is based on posterior mean estimates.

In conclusion, we would like to thank you and the reviewers for providing constructive criticism and making valuable suggestions. We believe that your comments have helped us to improve our study. In addressing the comments above and in the original manuscript, we hope that this is now to your satisfaction.

Sincerely,

Gianluca Boo and co-authors
