# Supplementary material for: Tackling public health data gaps through Bayesian high-resolution population estimation: A case study of Kasaï-Oriental, Democratic Republic of the Congo
Source: PLOS Glob Public Health. 2025 Sep 4;5(9):e0005072. doi: 10.1371/journal.pgph.0005072 (PMC12410768; doi:10.1371/journal.pgph.0005072)
Supplement: Attachment — Submitted filename: Review_2025_06_20.pdf [file pgph.0005072.s004.pdf]

Review for Revision of "Tackling public health data gaps through Bayesian high-resolution population estimation: a case study of Kasaï-Oriental, Democratic Republic of the Congo"

I thank the authors for addressing my comments on their original submission. While the revised manuscript is an improvement is many aspects, I have additional concerns which I enumerate below.

- Reviewer 1 previously commented that something like $s[i]$ is needed to indicate that settlement class is determined by the grid cell. I will defer to Reviewer 1 on their interpretation, but I agree that in Equations like Equation (11), $s[j]$ is necessary since the grid cell is indexed by j (matching the manuscript text of "each settled grid cell j is assigned to a settlement class"). I believe that $s[j]$ is not, however, required when specifying the priors.
- Equations (6) and (9) are missing the distributions for their respective β's.
- The procedure outlined in the "Gridded predictions" section starting on page 10 (or again in "Areal aggregations" on page 11) is not consistent with the Bayesian paradigm. Based on the text, it appears all estimates are drawn from posterior means (indicated by the hats) rather than on the posterior sample scale. However, based on the provided code, it appears the posterior samples may actually have been used. If the posterior means were used, then I believe the inference is not valid and the analysis should be redone with posterior samples. If the posterior samples were used, then I recommend rewriting the text to make this clear.
- It's no longer clear to me what Equation (1) contributes to the proposed model. The Poisson distribution appears to only introduce additional variation into the final estimates obtained from the building count and the population density model. Based on the models, the population estimates should come from either $B_i \cdot \lambda_i^{(D)}$ or $\lambda_i^{(B)} \cdot \lambda_i^{(D)}$ (where the multiplication happens on the posterior sample level), since these are the parameters estimated from data. The model in Equation (1) is never fit. While this approach could be used to introduce additional necessary variation, it would seem more appropriate to just allow more variation in $\lambda_i^{(P)}$ and use that directly as the estimate. If $\lambda_i^{(B)}$ was replaced with posterior predictive draws from Equation (3), that would bring the variation in building count downstream.
- Expanding on the point above, I'm confused by the fact that the building count data and parameters are multi-layered (observed B come from Poisson, and the mean comes from a log-normal), whereas the population density data and parameters are single layered (observed $\lambda_i^{(D)}$ come from log-normal). This creates a problem in Equation (2), where $\lambda_i^{(B)}$ is a parameter that was estimated (good), while $\lambda_i^{(D)}$ is the observed density data (problematic). One solution is to replace with $\lambda_i^{(D)}$ in Equation (2) with the estimated expected value of the population density, or to replace both with posterior predictive draws from their respective distributions.
- Equation (10) replaces the previously defined λ's in Equation (1) with $\hat{B}$ and $\hat{D}$. This should be rewritten to stay consistent (although $\hat{B}$ and $\hat{D}$ may be more appropriate above instead, as discussed in the previous two comments).

- Line 239 calls $\hat{\beta}_s^{(B)}$ random effects, but I believe these are now just settlement-class-specific fixed effects.
- The metrics for bias, imprecision, and inaccuracy seem very untraditional to me, and it's not clear they are valid. For example, the mean of the residuals for a linear model are 0, which does not indicate lack of bias. I'm not familiar with any literature that uses the mean of residuals for a Poisson model to evaluate bias.
- The Bulk Effective Sample Size (ESS) was reported on Line 290, but it's unclear what this sample size is with respect to. Is this the minimum ESS across the parameters from both models?
- Starting on Line 328, were any of the prediction-versus-observed checks performed for out-of-sample observations?
- The population count plot in Figure 3 seems to indicate an almost constant predicted value for the Urban settlement class, which leads to overestimation of small counts and underestimation of large counts. While some of this behavior was discussed in the main text, I'd appreciate additional discuss about causes of and the effects of this trend.
- Minor Comment: The switch from Greek letters to the spelling of Alpha, Sigma, and Beta seems odd to me.
