# Supplementary material for: Tackling public health data gaps through Bayesian high-resolution population estimation: A case study of Kasaï-Oriental, Democratic Republic of the Congo
Source: PLOS Glob Public Health. 2025 Sep 4;5(9):e0005072. doi: 10.1371/journal.pgph.0005072 (PMC12410768; doi:10.1371/journal.pgph.0005072)
Supplement: Attachment — Submitted filename: manuscript_review_2_respone.pdf [file pgph.0005072.s005.pdf]

Gianluca Boo

School of Geography and Environmental Science
University of Southampton, Southampton, UK

gianluca.boo@soton.ac.uk

21/07/2025

Manuscript Revision

Dear Dr Wheldon,

Thank you for the opportunity to submit a revised version of our manuscript PGPH-D-24-02971R1 entitled "Tackling Public Health Data Gaps through Bayesian High-Resolution Population Estimation: A Case Study of Kasaï-Oriental, Democratic Republic of the Congo." We greatly appreciate the time and effort that you and the reviewers have devoted to revising our work. We are pleased by the overall positive feedback and have carefully considered the insightful suggestions of Reviewer 3. We have addressed each of Reviewer 3's comments in detail with changes highlighted within the manuscript. Below, you will find our point-by-point response.

Reviewer 3:

I thank the authors for addressing my comments on their original submission. While the revised manuscript is an improvement is many aspects, I have additional concerns which I enumerate below.

We thank the reviewer for their time and the thoughtful, detailed feedback provided throughout the review process. We were pleased to revise the manuscript considering the initial comments and found the suggestions constructive. Below, we respond point-by-point to the additional concerns raised.

• Reviewer 1 previously commented that something like s[i] is needed to indicate that settlement class is determined by the grid cell. I will defer to Reviewer 1 on their interpretation, but I agree that in Equations like Equation (11), s[j] is necessary since the grid cell is indexed by j (matching the manuscript text of "each settled grid cell j is assigned to a settlement class"). I believe that s[j] is not, however, required when specifying the priors.

In our original response to Reviewer 1, we noted that parameters such as $\sigma_s^{(B)}$, $\alpha_s^{(B)}$, and $\beta_s^{(B)}$ are not indexed because they are shared across all clusters within settlement class s. However, we agree with Reviewer 3 that in the "Gridded predictions" section, specifically in Equations (10-13), formerly Equations (11-14), it is necessary to index s by j. We have revised these equations by replacing s with s_j to make clear that the settlement class varies with settled grid cell j. We have also updated the sentences at lines 233–236, 238-240, 242-244, and 246-248 to explain that while $\sigma_{sj}^{(B)}$ and $\alpha_{sj}^{(B)}$ remain settlement-class-specific, the class assignment s_j is indexed by settled grid cell.

• Equations (6) and (9) are missing the distributions for their respective β's.

Thank you for noticing this omission. We have now specified the prior distributions for β in Equation (5), formerly Equation (6), and Equation (8), formerly Equation (9), reporting the use of non-informative priors. We have also clarified in the text at lines 200-201 with reference at lines 219-221 that the choice of prior distributions is meant to encode minimal prior information about the parameters.

• The procedure outlined in the "Gridded predictions" section starting on page 10 (or again in "Areal aggregations" on page 11) is not consistent with the Bayesian paradigm. Based on the text, it appears all estimates are drawn from posterior means (indicated by the hats) rather than on the posterior sample scale. However, based on the provided code, it appears the posterior samples may actually have been used. If the posterior means were used, then I believe the inference is not valid and the analysis should be redone with posterior samples. If the posterior samples were used, then I recommend rewriting the text to make this clear.

We thank the reviewer for highlighting this crucial point. Indeed, all gridded and areal predictions were generated by propagating the full posterior sample, rather than by plugging in posterior means so that uncertainty in both parameters and latent variables is correctly reflected. We have now removed the stray "hats" in Equations (9–14) and the surrounding text.

• It's no longer clear to me what Equation (1) contributes to the proposed model. The Poisson distribution appears to only introduce additional variation into the final estimates obtained from the building count and the population density model. Based on the models, the population estimates should come from either $B_i \cdot \lambda_i^{(D)}$ or $\lambda_i^{(B)} \cdot \lambda_i^{(D)}$ (where the multiplication happens on the posterior sample level), since these are the parameters estimated from data. The model in Equation (1) is never fit. While this approach could be used to introduce additional necessary variation, it would seem more appropriate to just allow more variation in $\lambda_i^{(P)}$ and use that directly as the estimate. If $\lambda_i^{(B)}$ was replaced with posterior predictive draws from Equation (3), that would bring the variation in building count downstream.

We apologise for the confusion. In our implementation, we did indeed model the observed population P_i using a Poisson distribution with rate $\lambda_i^{(P)}$ computed as the product of $\lambda_i^{(B)}$ and $\lambda_i^{(D)}$. However, Equation (1) was not directly reflected in the Stan code because, for compacity's sake, we substituted $\lambda_i^{(B)} \cdot \lambda_i^{(D)}$ directly in place of $\lambda_i^{(P)}$ (see line 80 in the "model" block, and line 111 in the "generated quantities" in the Stan code). Thus, Equation (1) was never fit separately, which led to the apparent disconnect. We have now merged the original Equations (1) and (2) into a single, explicit statement of the Poisson model, now presented as Equation (1), so that the manuscript matches the Stan code exactly. We chose to retain the Poisson layer rather than reporting only $B_i \cdot \lambda_i^{(D)}$ or $\lambda_i^{(B)} \cdot \lambda_i^{(D)}$ because it properly captures the additional sampling variability in the observed population counts P_i. Also, we used $\lambda_i^{(B)}$ rather than B_i, to reflect our treatment of B_i as an error-prone observation of the "true" building count $\lambda_i^{(B)}$. We have completed the paragraph at lines 176-182 to reflect these considerations.

• Expanding on the point above, I'm confused by the fact that the building count data and parameters are multi-layered (observed B come from Poisson, and the mean comes from a log-normal), whereas the population density data and parameters are single layered (observed λ(D)i come from log-normal). This creates a problem in Equation (2), where λ(B)i is a parameter that was estimated (good), while λ(D)i is the observed density data (problematic). One solution is to replace with λ(D)i in Equation (2) with the estimated expected value of the population density, or to replace both with posterior predictive draws from their respective distributions.

Thank you for pointing out this additional source of confusion. In Equation (1), formerly Equation (2), $\lambda_i^{(D)}$ and $\lambda_i^{(B)}$ are not observed data but latent parameters defined through multi-layered processes. In our manuscript, we emphasised this difference using Latin characters to denote observed data and Greek

characters to denote latent parameters. We have completed the sentence at lines 176-179 to clarify that $\lambda_i^{(D)}$ and $\lambda_i^{(B)}$ are modelled rather than observed data.

• Equation (10) replaces the previously defined λ's in Equation (1) with B^ and D^. This should be rewritten to stay consistent (although B ˆ and D^ may be more appropriate above instead, as discussed in the previous two comments).

Thank you for the suggestion. We have updated Equations (9), (10), and (12), formerly Equations (10), (11), and (13), and the surrounding text by replacing B ˆ and D ˆ with $\lambda_j^{(B)}$ and $\lambda_j^{(D)}$, respectively.

• Line 239 calls β(B)s random effects, but I believe these are now just settlement-class- specific fixed effects.

We agree with the reviewer's suggestion. The coefficients $\beta_s^{(B)}$ are indeed settlement-class-specific effects rather than random effects. Accordingly, we have updated the wording in the sentence at lines 238-240 to refer to "settlement-class-specific effects".

• The metrics for bias, imprecision, and inaccuracy seem very untraditional to me, and it's not clear they are valid. For example, the mean of the residuals for a linear model are 0, which does not indicate lack of bias. I'm not familiar with any literature that uses the mean of residuals for a Poisson model to evaluate bias.

We thank the reviewer for raising this important point. While it is true that OLS residuals sum to zero by definition, our hierarchical model involves a more complex combination of data, parameters, and processes, which can introduce systematic over- or under-prediction at different levels. To quantify and communicate systematic error, we adopt the bias, imprecision, and inaccuracy metrics introduced by Leasure et al. (2020), which were used to account for uncertainty from all model components (i.e. parameter uncertainty, Poisson process error, and log-normal process error). We have extended the sentence at lines 272-276 to justify their use in our context with reference to Leasure et al. (2020) and expanded the caption of Table 2 at lines 365–369 to better define these metrics.

• The Bulk Effective Sample Size (ESS) was reported on Line 290, but it's unclear what this sample size is with respect to. Is this the minimum ESS across the parameters from both models?

Indeed, the Bulk ESS refers to the minimum ESS across the parameters in both the building-count and population-density models. Reporting the minimum ESS ensures that every parameter achieves adequate mixing and reliable posterior estimates since if even one parameter has low ESS, it could compromise inference. We have completed the sentences at lines 291-292 to clarify this point.

• Starting on Line 328, were any of the prediction-versus-observed checks performed for out-of-sample observations?

*We indeed conducted prediction-versus-observed checks for both in-sample and out-of-sample data. The results are available together with the model code and results on Zenodo, as reported as lines 358-360, in the population_model/model_evaluate directory. Files ending in "_full" correspond to in-sample evaluations, while those ending in "_crossValidation" contain the out-of-sample results. We have added a sentence at lines 360–362 to read: "These results are presented in two separate files for in-sample (*_full) and out-of-sample (*_crossValidation) data in the population_model/model_evaluate directory."*

• The population count plot in Figure 3 seems to indicate an almost constant predicted value for the Urban settlement class, which leads to overestimation of small counts and underestimation of large counts. While some of this behavior was discussed in the main text, I'd appreciate additional discuss about causes of and the effects of this trend.

We thank the reviewer for highlighting the predictive behaviour in urban settlements. This results from the fact that the population-density covariates explain relatively little variation beyond the settlement-class intercepts (see Figure 2). This means that weak covariate effects cause posterior population density estimates to "shrink" toward those intercepts, compressing the spread of predicted counts and, consequently, overestimating small population counts while underestimating large ones. We have added a sentence to discuss this issue in the Discussion section at lines 444-446 and 449-450.

• Minor Comment: The switch from Greek letters to the spelling of Alpha, Sigma, and Beta seems odd to me.

We thank the reviewer for pointing this out. We have reviewed the manuscript and revised all instances where Greek characters (e.g. α, β, σ) were spelt out as "alpha," "beta," or "sigma" in the main text and Figure 2.

We thank again the Editor and the reviewers for their thoughtful and constructive feedback, which has helped us clarify and strengthen the manuscript. We hope that the revisions and clarifications provided address all concerns.

Sincerely,

Gianluca Boo and co-authors
